# Research progress and application prospects of multi-omics integration strategies in precision risk stratification of type 1 diabetes mellitus

**DOI:** 10.3389/fimmu.2026.1891938

**Published:** 2026-08-19

**Authors:** Ziyi Chen, Wentao Liu, Zheng Guo, Yuying Guo, Jiaxin Hou, Mengpeng Rong, Huijuan Zheng, Zhaoli Cui

**Affiliations:** 1 Department of Nephrology and Endocrinology, Dongzhimen Hospital, Affiliated to Beijing University of Chinese Medicine, Beijing, China; 2 Department of Physiology, Pharmacology and Therapeutics, Johns Hopkins University School of Medicine, Baltimore, MD, United States; 3 Renal Research Institution of Beijing University of Chinese Medicine, Beijing, China

**Keywords:** biomarkers, early warning, individualized prevention, multi-omics integration, risk stratification, type 1 diabetes

## Abstract

Type 1 diabetes (T1D) is a chronic metabolic disease mediated by autoimmunity. Its pathogenesis involves complex interactions between genetic susceptibility and environmental factors. Conventional T1D risk stratification primarily relies on genetic markers, islet autoantibodies, and glycemic indicators. Although these biomarkers remain indispensable in current clinical practice, they are often insufficient when used alone to accurately identify ultra-early high-risk individuals, predict disease progression rates, or support individualized preventive strategies. Consequently, more comprehensive molecular approaches are needed to improve precision risk stratification. In recent years, the rapid development of multi-omics technologies has provided new strategies for precise risk stratification of T1D. This narrative review critically evaluates how multi-omics integration strategies can improve precision risk stratification throughout the T1D disease continuum by integrating complementary molecular information from genomics, transcriptomics, proteomics, metabolomics, epigenomics, and the microbiome. Particular emphasis is placed on stage-specific biomarker discovery, multi-omics data integration frameworks, artificial intelligence-assisted prediction models, biomarker validation, and the opportunities and challenges associated with clinical translation. Current evidence suggests that integrated multi-omics approaches have the potential to improve risk prediction accuracy, distinguish heterogeneous disease trajectories, identify individuals at imminent risk of progression, and provide biologically informed targets for precision intervention. However, important challenges remain, including data harmonization, external validation, model interpretability, cost-effectiveness, and integration into routine clinical screening programs. Future research should prioritize prospective multicenter cohorts, standardized analytical pipelines, externally validated prediction models, and clinically interpretable multi-omics frameworks to facilitate the translation of precision risk stratification into routine T1D prevention and management.

## 1 Introduction

Type 1 diabetes mellitus (T1D) is a chronic autoimmune disease characterized by progressive immune-mediated destruction of pancreatic β-cells, ultimately leading to lifelong insulin dependence. Despite substantial advances in insulin replacement therapy and glucose monitoring technologies, T1D continues to impose a considerable burden on patients and healthcare systems because of its lifelong management requirements and the risk of acute and chronic complications (1). Importantly, disease progression is highly heterogeneous among individuals, with marked differences in the timing of autoimmunity, β-cell decline, and clinical onset. This biological heterogeneity highlights the need for accurate risk stratification to identify individuals at different stages of disease progression and to facilitate timely preventive intervention.

T1D is now recognized as a progressive disease that evolves through well-defined preclinical and clinical stages rather than presenting as an abrupt onset of hyperglycemia. According to the current staging model, genetically susceptible individuals may first develop islet autoimmunity (Stage 1), followed by dysglycemia (Stage 2), before progressing to symptomatic diabetes (Stage 3). Throughout this disease continuum, distinct molecular alterations occur before measurable metabolic abnormalities become apparent. Consequently, the biomarkers required for risk assessment differ across disease stages, ranging from genetic susceptibility markers during early disease initiation to immune, proteomic, metabolic, and functional biomarkers that reflect ongoing β-cell injury and disease progression. This stage-specific biological evolution provides the rationale for integrating complementary omics data to achieve more precise and individualized risk stratification.

Early identification of individuals at high risk for T1D, together with targeted screening and timely preventive intervention, represents a key strategy for delaying disease progression, preserving β-cell function, and reducing the burden of disease and its complications.

Current T1D risk stratification primarily relies on established clinical markers, including HLA genotyping, islet autoantibodies, blood glucose measurements, HbA1c, and oral glucose tolerance testing (2). These markers have substantially improved early disease detection and remain the foundation of current screening strategies. However, when used individually, they provide only partial information regarding disease susceptibility, immune activation, β-cell injury, or metabolic dysfunction and therefore may not fully capture the biological heterogeneity or progression dynamics of T1D. Consequently, although these conventional markers remain indispensable in clinical practice, their limited ability to comprehensively characterize the multiple biological processes underlying T1D highlights the need for complementary molecular approaches capable of improving individualized risk stratification.

Recent advances in high-throughput multi-omics technologies have enabled comprehensive characterization of the diverse molecular alterations that occur throughout T1D development. Rather than providing isolated biological observations, complementary information obtained from genomics, transcriptomics, proteomics, metabolomics, epigenomics, and microbiome profiling can be integrated to construct a more comprehensive understanding of disease susceptibility, immune dysregulation, β-cell dysfunction, and metabolic progression (3). In this narrative review, we critically evaluate how multi-omics integration strategies can improve precision risk stratification across the T1D disease continuum. We focus on how complementary molecular information can be integrated with conventional clinical markers to enhance disease prediction, characterize disease heterogeneity, and support precision prevention. We also discuss data integration methods, AI-assisted prediction models, biomarker validation, and key barriers to clinical translation. By establishing a conceptual framework linking stage-specific molecular alterations with integrated risk prediction models, this review provides a comprehensive perspective on the opportunities and challenges of translating multi-omics integration into precision prevention and personalized management of T1D.

## 2 Clinical significance of T1D risk stratification and limitations of conventional markers

### 2.1 The core significance of T1D risk stratification

Type 1 diabetes mellitus (T1D) risk stratification refers to the classification of individuals into different risk categories based on genetic susceptibility, immune status, metabolic features, and clinical indicators. T1D is now recognized as a staged autoimmune disease. Stage 1 is defined by the presence of two or more islet autoantibodies with normoglycemia; stage 2 by multiple islet autoantibodies with dysglycemia but without overt symptoms; and stage 3 by symptomatic clinical diabetes (4). Therefore, risk stratification is not only a tool for identifying high-risk individuals, but also a framework for monitoring disease progression, guiding preventive intervention, and optimizing long-term management. The clinical significance of T1D risk stratification can be summarized in the following aspects.

#### 2.1.1 Identification of high-risk individuals for early prevention

T1D develops through a prolonged preclinical phase that may extend over several years or even decades, progressing from genetic susceptibility to islet autoimmunity and eventually to symptomatic diabetes. Risk stratification enables the identification of individuals at high risk during the preclinical stages of disease, thereby providing an opportunity for timely surveillance and preventive intervention. Risk stratification also facilitates stage-specific preventive strategies, including primary prevention before the onset of autoimmunity, secondary prevention following islet autoantibody seroconversion, and tertiary prevention after clinical diagnosis. Large prospective studies, such as TrialNet, have demonstrated that systematic screening and longitudinal monitoring of high-risk individuals enable earlier intervention and improve opportunities for preserving β-cell function (5).

#### 2.1.2 Disease staging and individualized monitoring

Because disease progression varies substantially among individuals, risk stratification enables stage-specific monitoring and individualized follow-up strategies. For individuals with rapid progress, the follow-up intensity can be strengthened; for those with slow progress, overtreatment can be avoided.

#### 2.1.3 Treatment optimization and prognosis assessment

Even after the clinical onset of the disease, risk stratification remains valuable. By evaluating the residual function of the patient’s beta-cells, the risk of complications and other factors, the insulin treatment plan can be adjusted individually, and the screening of complications can be carried out in a targeted manner, so as to improve the long-term outcomes. At the same time, after understanding the genetic factors affecting the function of beta-cells, the personalization of diabetes management can be realized, especially for those with high genetic risk of complications. The ISPAD 2024 guideline also incorporates treatment strategies for retaining beta-cell function, emphasizing the approach of preserving beta-cell function in patients with stage 3 T1D (6).

#### 2.1.4 Reduction of healthcare burden and resource utilization

Early identification and timely intervention have the potential to delay disease progression, preserve endogenous β-cell function, reduce diabetes-related complications, and improve long-term health outcomes. Health economic analyses further indicate that improving glycemic control through earlier intervention substantially increases quality-adjusted life years while reducing long-term healthcare costs (7), as summarized in **Table 1**.

**Table 1:** Lifetime health and economic benefits of improved glycemic control.

Level	Glycemic-control scenario	QALY gain	Cost savings
Patient	HbA1c: 8.0% to 7.0%	0.66 per patient	—
Patient	HbA1c: 9.0% to 7.0%	1.37 per patient	—
Population	Immediate vs. 1-year delay	9,438	€224 million
Population	Immediate vs. 3-year delay	27,171	€556 million
Population	Immediate vs. 7-year delay	72,717	€1.3 billion

HbA1c, glycated hemoglobin; QALY, quality-adjusted life year. Data are based on Moes et al. (2023).

### 2.2 Traditional T1D risk stratification method and limitations

Current clinical risk stratification for T1D primarily relies on three categories of biomarkers: genetic markers, islet autoantibodies, and metabolic indicators. Together, these markers form the foundation of current screening and disease staging strategies, enabling the identification of genetically susceptible individuals, detection of islet autoimmunity, and monitoring of glycemic deterioration. However, each biomarker category reflects only one aspect of disease biology. Consequently, when used independently, they may be insufficient to capture the dynamic molecular changes underlying disease initiation and progression, limiting their ability to support individualized risk prediction and precision prevention.

#### 2.2.1 Genetic indicators

Genetic susceptibility constitutes the earliest identifiable risk factor for T1D.Over half of the genetic risk has been attributed to the human leukocyte antigen (HLA) class II gene region and to the insulin (INS) gene locus – both thought to confer direction of autoreactivity and tissue specificity (8). A review published in Current Diabetes Reports in 2025 further confirmed that more than 90 genetic loci have been associated with T1D risk, of which about half of the genetic risks come from HLA sites, and non-HLA sites have less impact on disease risk (2). However, because genetic variants remain largely static throughout life, genomic information alone cannot reflect dynamic immune activation, β-cell injury, or metabolic deterioration. Therefore, genomic information is most informative when integrated with dynamic molecular biomarkers, such as transcriptomic, proteomic, and metabolomic profiles, to improve individualized risk prediction.

#### 2.2.2 Immune indicators

Islet autoantibodies are central immune markers for the diagnosis and risk assessment of clinical type 1 diabetes. The appearance of autoantibodies marks the initiation of autoimmune response and is an important reference indicator for T1D diagnosis (4). At present, the main islet autoantibodies for routine clinical detection include five types: glutamate decarboxylase antibody (GADA), insulin autoantibody (IAA), pancreatic islet cell antibody (ICA), protein tyrosine phosphatase antibody (IA-2A) and zinc transporter 8 antibody (ZnT8A) (9, 10). Multiple positive antibodies (≥2) indicate a higher risk of onset. However, these indicators have limitations. Some high-risk groups continue to show negative antibodies, leading to significant underdiagnosis. The level of autoantibody itself also fluctuates over time. A study published in Diabetes in 2024 found that among young people who progressed to clinical T1D, 39% of the multi-antibody positive people in the DAISY cohort and 10% of the ASK screening study returned to a single antibody or negative state before clinical diagnosis, while up to 30% of stage 2 T1D patients It is presented as a single antibody or negative state in a single screening (11). The positive rate of antibodies is affected by age, testing methods, ethnicity, and other factors, and there are large individual differences (12). Antibody titre, the review points out that islet autoantibodies usually appear in the early life, and their quantity and titer may fluctuate for more than 20 years before the onset of T1D. Although the increase in the number of antibodies seems to suggest that the disease is progressing towards clinical T1D, the antibody titre changes themselves cannot provide real-time information on the decline of beta-cell function, nor can it accurately distinguish between fast progressors and slow progressors (13). The progression risk difference in multi-antibody positive population is still large. Although islet autoantibodies remain the gold standard for identifying islet autoimmunity and defining Stage 1 and Stage 2 T1D, antibody positivity alone cannot fully explain the marked inter-individual variability in disease progression. Integrating autoantibody status with complementary molecular biomarkers may improve prediction of disease trajectory.

#### 2.2.3 Clinical indicators

Blood glucose-related indicators are the core of T1D diagnosis and monitoring, including fasting plasma glucose (FPG), oral glucose tolerance test (OGTT) 2-hour blood glucose, glycated hemoglobin (HbA1c), etc. In recent years, indicators such as coefficient of glycemic variation and time in range(TIR) derived from continuous blood glucose monitoring (CGM) have also been used to evaluate the quality of glycemic control (14). The doctoral thesis of the University of Bristol in the United Kingdom confirmed that the hallmark feature of T1D is an immune-mediated decrease in pancreatic islet beta-cell mass, “when about 70%~80% of beta-cells are destroyed, it leads to high blood glucose”, therefore, blood glucose-related indicators reflect the metabolic state after the disease occurs (15). At this time, the disease has entered an irreversible stage. It is impossible to achieve real early warning by relying on clinical indicators, missing the best intervention window period, and cannot be used in the first-level prevention stage. Risk stratification. Even after diagnosis, HbA1c has inherent limitations as the “gold standard” for evaluating glycemic control. A study published in the World Journal of Diabetes in 2025 clearly pointed out that “HbA1c has limited ability to reflect the risk of hypoglycemia and blood glucose variability, which is particularly worrying in patients with T1D” (16). In addition, Blood glucose-related indicators have limited ability to predict the progression rate of T1D. The special review published by Lancet Diabetes & Endocrinology in 2024 pointed out that although abnormal blood glucose (stage 2) marks the disease entering the stage of metabolic deterioration, there is a high degree of heterogeneity in the progress rate of different individuals, based on blood glucose and HbA1c alone. The method distinguishes between rapid progressors and slow progressors, and it is impossible to accurately predict when to enter the clinical stage 3 (17). Even after the diagnosis, the relationship between the level of glycemic control and the decline rate of beta-cell function and the risk of complications is complex, and it is difficult to achieve individualized prognosis assessment by relying on blood glucose indicators alone. Glycemic indicators accurately reflect metabolic deterioration but generally become abnormal only after substantial β-cell dysfunction has already occurred, limiting their value for ultra-early risk prediction.

In summary, conventional clinical markers remain indispensable for T1D screening, disease staging, and clinical management. However, because each biomarker reflects only a single dimension of disease biology, no individual marker can comprehensively characterize the complex interactions among genetic susceptibility, immune activation, β-cell dysfunction, and metabolic alterations. These limitations provide a strong rationale for integrating complementary molecular information from genomics, transcriptomics, proteomics, metabolomics, epigenomics, and microbiome profiling. Such multi-omics integration offers the potential to achieve more accurate, dynamic, and individualized risk stratification throughout the T1D disease continuum (3).

## 3 Contributions of individual omics layers to T1D risk stratification

Individual omics layers provide complementary information for understanding T1D susceptibility, immune activation, β-cell stress, metabolic dysregulation, and disease progression. Although each omics approach has independent value, no single layer can fully capture the biological heterogeneity of T1D. Therefore, this section summarizes the contribution, clinical relevance, and limitations of each omics layer as the foundation for subsequent multi-omics integration.

### 3.1 Genomics: inherited susceptibility and early risk identification

Genomics provides the earliest and most stable layer of information for T1D risk stratification by identifying inherited susceptibility before the onset of islet autoimmunity. Genome-wide association studies have shown that T1D is a highly polygenic disease, with more than 90 susceptibility loci identified to date. Among these, the HLA class II region represents the strongest genetic determinant, accounting for a substantial proportion of inherited risk. HLA-DR and HLA-DQ alleles influence antigen presentation and shape autoreactive T-cell responses, making HLA genotyping a cornerstone of genetic risk assessment (18).

#### 3.1.1 HLA II gene - the most important susceptibility genes

HLA genotyping has been widely used in risk prediction and neonatal screening studies to identify individuals who may benefit from longitudinal autoantibody surveillance. However, HLA risk alone is insufficient for individualized prediction. A considerable proportion of individuals carrying high-risk HLA haplotypes never develop T1D, whereas more than 10% of patients with T1D do not carry the classic high-risk HLA-DR3 or HLA-DR4 haplotypes. These observations highlight the importance of non-HLA loci and downstream molecular events in determining disease progression (19).

#### 3.1.2 Non-HLA susceptibility gene

Non-HLA susceptibility loci, including INS, PTPN22, CTLA4, and IL2RA, further contribute to T1D risk by regulating insulin antigen expression (20). T-cell receptor signaling, immune checkpoint function, and regulatory T-cell homeostasis. These loci help explain part of the heterogeneity in immune tolerance and disease susceptibility. Nevertheless, their individual effect sizes are generally modest, and their predictive value is strongest when incorporated into polygenic or genetic risk scores.

#### 3.1.3 Role and limitations of genomics in T1D risk stratification

##### 3.1.3.1 Main functions: identify high-risk populations of genetics

Genomic information is particularly valuable for identifying individuals with inherited susceptibility before the development of islet autoimmunity. HLA and non-HLA genotyping, as well as genetic risk scores, can be applied at birth or in early childhood to guide longitudinal surveillance and determine the frequency of islet autoantibody screening. For example, neonatal screening studies such as Freder1k have demonstrated the feasibility of using selected genetic variants to identify infants at increased risk and invite them for scheduled autoantibody follow-up (21). In addition, certain loci, such as INS and PTPN22, may contribute to differences in disease progression and age at onset (22).

However, the major limitation of genomics is that it reflects static inherited susceptibility rather than dynamic disease activity. Genotypes remain constant throughout life and cannot capture environmental triggers, immune activation, β-cell stress, metabolic deterioration, or the timing of progression from genetic risk to clinical diabetes (23). Moreover, genetic risk scores may have reduced performance across diverse ethnic populations and cannot independently predict the precise onset or progression rate of T1D. Therefore, genomic risk should be viewed as a foundational layer for early risk identification rather than a standalone stratification tool. Its predictive value is likely to improve when integrated with dynamic biomarkers, including islet autoantibodies, transcriptomic signatures, proteomic markers, metabolomic profiles, and microbiome-related environmental information (24).

### 3.2 Transcriptomics: dynamic immune activation and β-cell stress signatures

Transcriptomics provides a dynamic view of gene expression changes associated with immune activation, β-cell stress, and disease progression in T1D. Unlike genomics, which reflects inherited susceptibility, transcriptomic profiling can capture stage-specific molecular activity in peripheral blood immune cells, pancreatic islets, and β-cells exposed to inflammatory stress (25, 26). Therefore, transcriptomics is particularly useful for identifying pathways that become activated after genetic risk has translated into immune dysregulation.

Peripheral blood transcriptomic studies have identified immune-cell signatures associated with T1D risk and progression, including interferon-stimulated genes, cytokine-response pathways, antigen presentation, and T-cell activation programs (27). These signatures may help distinguish individuals with active immune dysregulation from those with genetic susceptibility alone. In parallel, pancreatic islet and β-cell transcriptomic analyses provide insight into β-cell-intrinsic stress responses, including endoplasmic reticulum stress, inflammatory signaling, HLA class I upregulation, and apoptosis-related pathways.

Single-cell RNA sequencing has further improved the resolution of transcriptomic analysis by allowing cell-type-specific characterization of immune cells and pancreatic islet cells. This approach can distinguish transcriptomic changes in β-cells, α-cells, endothelial cells, and infiltrating immune populations, thereby clarifying how cellular heterogeneity contributes to T1D progression. In addition, non-coding RNAs, including microRNAs and long non-coding RNAs, may participate in immune regulation, β-cell dysfunction, and inflammatory responses, providing additional candidate biomarkers for disease monitoring.

However, transcriptomic biomarkers still face several limitations. Gene expression patterns are highly sensitive to tissue source, disease stage, inflammatory status, treatment exposure, and technical platform. Peripheral blood transcriptomic signatures may not fully reflect molecular events occurring within pancreatic islets, while access to human islet tissue is limited. Therefore, transcriptomic findings require longitudinal validation and integration with proteomic, metabolomic, epigenomic, and clinical data before they can be used reliably for precision risk stratification.

### 3.3 Proteomics:functional biomarkers for disease progression and risk stratification

Proteomics systematically characterizes protein expression and post-translational modifications in biological samples using high-throughput technologies, particularly mass spectrometry. As proteins are the direct executors of biological functions, proteomic profiling provides a functional link between genetic susceptibility and clinical phenotype. Compared with genomics and transcriptomics, proteomics more directly reflects immune activation, β-cell stress, inflammatory responses, and metabolic dysfunction during T1D progression, making it a valuable approach for biomarker discovery and precision risk stratification.

#### 3.3.1 Novel protein biomarkers for early risk prediction

Proteomic studies have identified several candidate biomarkers associated with disease progression before the onset of clinical diabetes. A study of children with rapidly progressive T1D reported that serum levels of two APOC1-derived peptides decreased shortly after islet autoantibody seroconversion and remained significantly lower than those in antibody-negative controls among individuals who subsequently developed clinical T1D (28). These findings suggest that APOC1 may serve as a candidate biomarker for identifying individuals at imminent risk of progression to clinical T1D, thereby facilitating earlier risk stratification and more timely preventive intervention. However, these findings require validation in larger prospective cohorts before routine clinical application.

#### 3.3.2 Proteomic biomarkers reflecting disease mechanisms

Beyond biomarker discovery, proteomics provides important mechanistic insights into β-cell dysfunction and immune-mediated injury. For example, protein disulfide isomerase A1 (PDIA1), an endoplasmic reticulum chaperone involved in protein folding and redox regulation, has been proposed as a biomarker of β-cell endoplasmic reticulum stress. Increased PDIA1 expression reflects early β-cell dysfunction before overt autoimmune destruction, providing mechanistic evidence linking proteomic alterations to disease initiation (29).

#### 3.3.3 Non-invasive proteomic biomarkers: revealing the molecular mechanism of pathogenesis:

Tear proteomics has recently emerged as a promising non-invasive strategy for T1D biomarker discovery, particularly for longitudinal monitoring in pediatric populations. Tears are readily accessible and contain abundant proteins, lipids, and metabolites. A large-scale LC-MS/MS study identified 3,302 tear proteins, of which 239 were differentially expressed between children with T1D and healthy controls (30, 31). These proteins were predominantly associated with immune responses, inflammation, and tissue homeostasis. Increased immunoglobulin and complement proteins together with reduced S100A8 and S100A9 expression suggest persistent ocular immune activation in T1D. Furthermore, tear proteomic profiles were influenced by diabetic ketoacidosis at diagnosis and glycemic control, indicating potential utility for monitoring disease progression and long-term complications.

#### 3.3.4 Clinical applications of proteomics in T1D risk stratification

Targeted and untargeted proteomic analyses have substantially expanded the repertoire of circulating biomarkers associated with T1D progression. A recent systematic review identified 266 unique serum and plasma proteins across 13 proteomic studies, with 31 proteins consistently replicated in multiple independent cohorts (32). These proteins were primarily involved in complement activation, lipid metabolism, and immune response pathways. Rather than relying on individual biomarkers, integrating multiple proteins into composite risk models has the potential to improve prediction accuracy and facilitate the transition from single-time-point assessment to dynamic disease monitoring.

#### 3.3.5 Proteomics for complication risk stratification

Proteomics also provides valuable biomarkers for predicting diabetes-related complications. In diabetic kidney disease, an eight-protein urinary biomarker panel demonstrated excellent diagnostic performance and significantly outperformed urinary albumin alone in distinguishing patients with and without nephropathy (33). For cardiovascular complications, elevated serum APOC3 and HDL-associated APOC3 were independently associated with future cardiovascular events in adults with T1D (34). In addition, serum neurofilament light chain (NFL), a marker of axonal injury, has been strongly associated with diabetic peripheral neuropathy and correlates with impaired nerve conduction and sensory dysfunction (35). These findings highlight the value of proteomics not only for disease prediction but also for long-term prognosis and complication surveillance.

#### 3.3.6 Role and limitations of proteomics in T1D risk stratification

Overall, proteomics provides functional biomarkers that directly reflect immune activation, β-cell stress, metabolic alterations, and tissue injury, making it particularly valuable for disease monitoring and complication risk stratification. Compared with genomic biomarkers, proteomic alterations are more closely associated with ongoing disease activity and therefore provide greater potential for dynamic risk assessment. However, widespread clinical implementation remains limited by the high cost of mass spectrometry, the lack of standardized protocols for sample collection and data analysis, and variability among analytical platforms. Furthermore, proteomic biomarkers should not be interpreted in isolation but rather integrated with genomic, transcriptomic, metabolomic, and clinical information to achieve more accurate and robust precision risk stratification.

### 3.4 Metabolomics:dynamic metabolic signatures for early risk stratification and disease monitoring

Metabolomics systematically characterizes small-molecule metabolites in biological samples, including serum, urine, saliva, and other biofluids, using high-throughput analytical platforms such as mass spectrometry (MS) and nuclear magnetic resonance (NMR) (36). As the downstream products of genomic, transcriptomic, and proteomic regulation, metabolites provide a direct functional readout of cellular physiology and metabolic homeostasis. Consequently, metabolomics reflects the combined effects of genetic susceptibility, immune activation, environmental exposure, and β-cell dysfunction. Importantly, metabolic alterations may occur before the appearance of islet autoantibodies and overt hyperglycemia, making metabolomics particularly valuable for ultra-early risk prediction, disease progression monitoring, and complication risk assessment (37).

#### 3.4.1 Early metabolic biomarkers preceding clinical disease

One of the major advantages of metabolomics is its ability to detect metabolic disturbances before the clinical onset of T1D. Longitudinal analyses from the DIPP (Type 1 Diabetes Prediction and Prevention) cohort demonstrated that children who subsequently developed multiple islet autoantibodies exhibited disturbances in secondary bile acid metabolism during infancy (38). Because bile acid metabolism is closely regulated by the gut microbiota, these findings suggest that disruption of the gut–islet axis may represent one of the earliest metabolic events in T1D pathogenesis. Therefore, bile acid profiles in serum or feces may provide biomarkers for identifying high-risk individuals before conventional immunological markers become detectable.

#### 3.4.2 Metabolic alterations associated with disease progression

Metabolomic profiling has identified characteristic metabolic signatures associated with T1D progression. One of the earliest reported abnormalities is altered choline metabolism. Umbilical cord serum lipidomic analysis demonstrated that children who later developed T1D exhibited significantly reduced levels of choline-containing phospholipids, including phosphatidylcholines and sphingomyelins, from birth (39). Importantly, these lipid alterations were specifically associated with progression to clinical T1D rather than the development of islet autoimmunity alone, suggesting that disturbed phospholipid metabolism represents an early disease-specific metabolic signature.

In addition, abnormalities in branched-chain amino acid (BCAA) metabolism have consistently been associated with T1D progression. Elevated circulating BCAA concentrations are commonly observed during preclinical stages and are associated with β-cell stress and metabolic dysfunction. As insulin deficiency progresses, BCAA levels may subsequently decline because of enhanced protein catabolism, reflecting increasing metabolic deterioration (37, 40). Collectively, these findings demonstrate that metabolomic profiling provides dynamic information regarding disease progression that cannot be obtained from static genetic markers alone.

#### 3.4.3 Metabolomics for complication risk stratification

Beyond predicting disease onset, metabolomics also contributes substantially to the risk stratification of diabetes-related complications. Prospective metabolomic studies have demonstrated that specific circulating metabolites are independently associated with diabetic nephropathy, retinopathy, and diabetic sensorimotor polyneuropathy (41). Incorporating metabolites such as 3,4-dihydroxybutyric acid, ribonic acid, and glycine into conventional clinical prediction models significantly improves the prediction of major microvascular outcomes.

Similarly, long-term follow-up studies of patients with T1D have shown that urinary metabolites, including branched-chain amino acids, pseudouridine, threonine, and citric acid, independently predict the progression of diabetic kidney disease beyond traditional clinical indicators such as albuminuria and estimated glomerular filtration rate (42). These findings highlight the clinical value of metabolomics for dynamic monitoring and individualized risk assessment of chronic complications.

#### 3.4.4 Clinical implications and therapeutic opportunities

Unlike genomic biomarkers, metabolomics not only identifies disease-associated biomarkers but also reveals potentially modifiable metabolic pathways. Altered bile acid metabolism links intestinal microbiota with host metabolism through activation of the farnesoid X receptor (FXR) and the G protein-coupled bile acid receptor TGR5. These pathways may be therapeutically modulated through dietary intervention, probiotics, prebiotics, or microbiome-directed therapies, providing novel opportunities for precision prevention and metabolic intervention in T1D (43).

#### 3.4.5 Role and limitations of metabolomics in T1D risk stratification

Overall, metabolomics provides dynamic functional information that reflects metabolic disturbances occurring throughout T1D development. Compared with genomics, metabolomic alterations are more responsive to environmental influences and disease activity, enabling earlier detection of metabolic dysfunction and improved prediction of disease progression and complications (44). However, metabolomic profiles are strongly influenced by diet, age, gut microbiota composition, and analytical platforms, leading to considerable inter-study variability. In addition, standardized analytical protocols and validation in large prospective cohorts remain necessary before metabolomic biomarkers can be routinely implemented in clinical practice. Therefore, metabolomics should be considered a complementary component of multi-omics integration rather than a standalone approach. Integrating metabolomic data with genomic, transcriptomic, proteomic, epigenomic, microbiome, and clinical information is expected to substantially improve the accuracy and robustness of precision risk stratification in T1D.

### 3.5 Epigenomics

Epigenomics studies the molecular process of gene expression through DNA methylation, histone modification, chromatin remodeling and other mechanisms without changing the DNA sequence (45). As a “molecular bridge” connecting genetic susceptibility and environmental factors, epigenetic modification can dynamically respond to environmental stimuli such as viral infection, dietary nutrition and intestinal flora, and play a key role in the pathogenesis of T1D. Unlike static genome sequences, epigenetic markers are reversible and environmentally responsive, providing more dynamic and stable predictive indicators for risk stratification.

Studies have found that there are multiple differential methylation regions (DMRs) in different immune cell subgroups between autoantibody-positive children and control groups.The core finding of the study is that through the analysis of samples before serum conversion, the DNA methylation characteristics in the very early stage of the disease were successfully identified (46).

#### 3.5.1 The unique advantages of epigenomics in risk stratification

##### 3.5.1.1 More stable early warning indicators than metabolome/proteogroup

Dang et al. Conducted whole-genome DNA methylation analysis of 24 pairs of T1D inconsistent monozygotic twin cells through Illumina 450K chip and hydrogen sulfite sequencing, confirming for the first time that methylation differences can exist stably for a long time, suggesting that DNA methylation can be used as a stable biomarker (47).

##### 3.5.1.2 Provide reversible intervention targets

The reversibility of epigenetic modification is its biggest advantage. Unlike genetic mutations, epigenetic changes can be reversed by drug intervention: DNA methyltransferase inhibitors (DNMTi): such as 5-azacytidine, decitabine, which can reverse abnormal high methylation and restore immunomodulatory group. Due to expression; histone deacetylase inhibitors (HDACi): such as valproic acid and sodium butyrate, which can adjust the level of histone acetylation, enhance Treg function, and inhibit the activation of T cells. At present, these drugs are mainly used for tumor treatment, and their application in T1D is still in the preclinical research stage, which requires strict clinical trials to verify their safety and effectiveness (48).

### 3.6 Microbiome and host-microbiome interactions

Microbiomics analyzes the composition and function of host symbiotic microorganisms (mainly intestinal flora) through high-throughput sequencing technology. As the “second genome”, the intestinal flora contains about 3 million genes, and its metabolites and immunomodulatory functions deeply affect the host’s metabolism and immune homeostasis. In T1D, dysbiosis is considered to be a key environmental factor linking genetic susceptibility and autoimmune activation.

#### 3.6.1 Characteristics of intestinal flora disorder in T1D

##### 3.6.1.1 Changes in the composition of the flora

Multiple case-controlled and longitudinal cohort studies (e.g. TEDDY, ABIS, DIPP) revealed changes in the characteristics of intestinal flora in T1D high-risk populations and patients.

The α-diversity of the flora is significantly reduced (49); the abundance of short-chain fatty acid (SCFA) bacteria is reduced; the conditional pathogenic bacteria increases;the function of the flora has changed;the number of lipopolysaccharides (LPS) produces more bacteria.

##### 3.6.1.2 Time dynamics: flora disorder precedes autoimmune activation

Longitudinal birth cohort studies show that intestinal flora disorder occurs before the emergence of pancreatic islet autoantibodies, suggesting that flora changes may be a promoting factor for autoimmunity rather than a result (50).

#### 3.6.2 Molecular mechanism of intestinal flora affecting the risk of T1D

##### 3.6.2.1 Immunomodulatory effect of short-chain fatty acids (SCFAs)

The main SCFAs (acetic acid, propionic acid, butyric acid) produced by fermented dietary fiber in intestinal flora are the key mediators connecting the flora and host immunity (51).

##### 3.6.2.2 Bacteria-intestinal-islet axis (gut-islet axis)

The intestinal flora affects the function of the islet through a variety of ways. The definition of the intestinal-pancreatic islet axis is “an important endocrine signal axis that regulates pancreatic islet function through the dialogue between intestinal microecology and endocrine metabolism”, and points out that the discovery of GLP-1, GIP and other intestinal insulin hormones has initially established a bridge between the intestine and pancreatic islet cells (52, 53).

#### 3.6.3 Application of microbiology in risk stratification

##### 3.6.3.1 Risk prediction model based on flora

Integrating the data of flora composition (16S rRNA sequencing) and flora metabolic function (metagenomic sequencing) can build a T1D risk prediction model.

##### 3.6.3.2 Geographical and crowd-specific stratification

There are significant differences in the characteristics of the flora of people in different regions, and it is necessary to establish a risk stratification standard for population specificity:

#### 3.6.4 Intervention strategy based on bacteria

The ultimate goal of microbiology is to develop operational intervention methods:

##### 3.6.4.1 Probiotics and prebiotics

- Specific strain supplement: Faecalibacterium prausnitzii, Akkermansia muciniphila, specific Bifidobacterium and Lactobacillus strains
- Prebiotic intervention: dietary fiber (e.g. oligofructose, inulin) promotes the growth of beneficial bacteria and the production of SCFA

##### 3.6.4.2 Fecal bacteria transplantation

Transplant the intestinal flora of healthy donors to T1D high-risk individuals or patients to rebuild a healthy flora ecosystem. At present, it is in the clinical trial stage, and it is necessary to solve problems such as donor screening, standardized scheme and long-term safety.

##### 3.6.4.3 Dietary intervention

High-fiber diet: increase the intake of fermentable dietary fiber and promote the production of SCFA; Mediterranean diet model: rich in polyphenols and dietary fiber, which is conducive to the diversity of bacterial flora; avoid over-processed food: reduce the negative impact of emulsifiers, artificial sweeteners, etc. on the flora (51).

#### 3.6.5 Limitations and prospects

Intestinal flora research still faces multiple challenges. The systematic review published in Cureus in 2024 pointed out that there is significant heterogeneity in the existing research design, sample size and analysis methods, which poses a challenge to the universality and explanatoryity of the results, and emphasizes the need for further research to clarify the mechanism between the flora and diabetes (54). In the future, it is necessary to combine multi-omic data (flora genome + metabolome + host immune phenotype) to establish an accurate “flora-host” interaction model to achieve individualized risk stratification and intervention based on microbiome. Taken together, individual omics layers capture distinct but complementary dimensions of T1D susceptibility and progression, with substantial differences in biological information, disease-stage relevance, analytical platforms, and readiness for clinical translation, as summarized in **Table 2**. Although each omics layer provides valuable stage-specific information, their complementary strengths and individual limitations provide a strong rationale for integrating multiple molecular layers within a unified risk-stratification framework.

**Table 2:** Representative evidence for omics layers in precision risk stratification of type 1 diabetes.

Omics layer	Representative cohort/study	Design/population	Specimen/platform	Stage mapped	Main markers/pathways	Validation status and clinical implication
Genomics	TrialNet, newborn-screening and GRS studies (7–12)	High-risk relatives, incident cases, newborn pilot cohorts	HLA, SNPs, GRS/PRS	Genetic susceptibility and screening entry	HLA-DR/DQ, INS, PTPN22, IL2RA; ancestry-aware PRS	Clinically feasible for enrichment; limited for timing of progression without dynamic markers
Transcriptomics	TEDDY and enterovirus-linked transcriptomic studies (13, 14)	Longitudinal childhood cohorts before seroconversion and T1D	Peripheral blood RNA-seq, immune-cell deconvolution	Pre-seroconversion to stage 1	Interferon signaling, monocyte/B-cell composition, IAA-first vs GADA-first programs	Mechanistically strong; requires replication and targeted panels for clinical use
Single-cell transcriptomics	Longitudinal CD4+ T-cell scRNA-seq (15)	At-risk or early-stage individuals sampled over time	Single-cell RNA-seq	Early immune activation and stage transition	T-cell activation, regulatory and memory programs	High resolution but costly; discovery and endotype development
Proteomics	TEDDY plasma proteins and systematic reviews (18, 19)	Longitudinal plasma and serum studies	LC-MS/MS, targeted proteomics, affinity platforms	Pre-autoimmunity, stage 1/2 progression	Complement, lipid transport, immune response, extracellular matrix	Promising for targeted panels; needs platform harmonization and external validation
Beta-cell stress proteomics	PDIA1 discovery proteomics (20)	Discovery cohorts and beta-cell stress models	Serum and mechanistic proteomics	Beta-cell stress before or during immune attack	Endoplasmic-reticulum stress, protein folding, redox regulation	Mechanistic marker; not yet clinical prediction tool
Metabolomics/lipidomics	TEDDY metabolome-wide and integrated infant analyses (22–24)	Longitudinal birth cohorts	LC-MS/MS, GC-MS, NMR, lipidomics	Pre-seroconversion and stage 1	Lipid remodeling, phospholipids, amino acids, metabolic stress	Potential early-warning layer; requires targeted validation
Bile acid metabolism	DIPP/T1D bile-acid studies (25)	Early-life longitudinal samples	Serum/fecal metabolomics	Before IA and T1D	Secondary bile acid dysregulation; gut-islet immune axis	Mechanistically plausible; causality and intervention remain unproven
Complication metabolomics	FinnDiane and microvascular studies (28, 29)	Large T1D complication cohorts	Urinary and circulating metabolites	Post-stage 3 prognosis	BCAAs, glycine, ribonic acid and DKD/retinopathy signals	Useful for whole-course risk stratification, not direct evidence for onset prediction
Epigenomics	DAISY, DIPP and methylome studies (30–34)	Longitudinal preclinical and maternal-exposure cohorts	DNA methylation, chromatin accessibility, single-nucleus assays	Pre-seroconversion to progression	Immune-cell methylation, chromatin priming, environmental memory	Promising but cell-composition and platform effects require careful control
Microbiome interface	TEDDY, early-life microbiome and new-onset studies (35–39)	Birth cohorts and case-control studies	16S, shotgun metagenomics, functional profiling	Environmental modifier across continuum	SCFA producers, microbial maturation, bile-acid and immune interactions	Context-dependent; distinguish association, reverse causation and causal mechanisms
Parallel multi-omics	TEDDY integration and high-risk subject studies (40, 46, 47)	Longitudinal or proof-of-concept multi-layer designs	Transcriptome, proteome, metabolome, lipidome	Preclinical and high-risk stages	Inflammation, lipid metabolism, T-cell/macrophage pathways	Supports integration but requires larger prospective validation

## 4 Multi-omics integration strategies for precision risk stratification in T1D

### 4.1 Rationale for multi-omics integration

Type 1 diabetes (T1D) is a heterogeneous autoimmune disease resulting from complex interactions among genetic susceptibility, immune dysregulation, environmental exposure, β-cell dysfunction, metabolic alterations, and gut microbiota. These biological processes occur across multiple molecular layers, including the genome, epigenome, transcriptome, proteome, metabolome, and microbiome. Each individual omics platform captures only one aspect of disease biology and therefore provides an incomplete representation of disease initiation and progression. For example, genomics identifies inherited susceptibility but cannot reflect dynamic immune activation, whereas metabolomics captures functional metabolic changes but cannot determine their upstream regulatory mechanisms.

Multi-omics integration overcomes these limitations by combining complementary molecular information across different biological layers to reconstruct regulatory networks from genotype to phenotype. Compared with single-omics analyses, integrated multi-omics approaches provide a more comprehensive understanding of disease heterogeneity, improve biomarker discovery, enhance prediction of disease progression, and support precision prevention and individualized intervention. Consequently, multi-omics integration has become one of the most promising strategies for precision risk stratification in T1D.

### 4.2 Strategies for multi-omics integration

Several computational strategies have been developed to integrate high-dimensional multi-omics datasets and extract biologically meaningful information.

#### 4.2.1 Statistical integration methods

Traditional statistical approaches remain fundamental tools for multi-omics analysis. Principal component analysis (PCA), partial least squares (PLS), and other multivariate dimension-reduction methods reduce data complexity while preserving major biological variation. Penalized regression approaches, including LASSO and Elastic Net, are widely used to identify the optimal combinations of biomarkers from high-dimensional datasets and construct parsimonious predictive models.

#### 4.2.2 Network-based integration

Network-based approaches aim to identify coordinated molecular interactions across different omics layers. Among these, weighted gene co-expression network analysis (WGCNA) is one of the most frequently applied methods. By integrating GWAS summary statistics with transcriptomic data, WGCNA identifies gene modules associated with specific biological pathways. For example, a study published in Genes & Immunity identified seven co-expression modules associated with T1D, with one module showing strong enrichment for immune regulatory pathways, thereby revealing core regulatory genes involved in disease pathogenesis (55).

#### 4.2.3 Machine learning and artificial intelligence

Artificial intelligence (AI) has substantially expanded the analytical capability of multi-omics integration by modeling complex nonlinear relationships that are difficult to capture using conventional statistical methods (56). Random Forest algorithms effectively identify important biomarker combinations and rank feature importance, whereas deep learning approaches, including autoencoders and graph neural networks, can integrate heterogeneous multi-omics datasets to identify latent molecular patterns and improve disease classification. These AI-based approaches are increasingly being incorporated into precision risk prediction models for T1D. More broadly, multi-omics integration strategies differ substantially in their analytical objectives, data requirements, interpretability, susceptibility to bias, and requirements for validation, as summarized in **Table 3**.

**Table 3:** Multi-omics integration and AI strategies for T1D risk stratification.

Strategy	Best suited data/question	Strengths	Key risks	Recommended reporting
Early feature-level integration	Harmonized, complete multi-omics with adequate sample size; prediction of IA or stage 3 onset	Simple pipeline; can maximize predictive discrimination	High-dimensional overfitting, batch effects, collinearity, platform bias	Nested cross-validation, external validation, calibration, decision-curve analysis
Intermediate latent-factor integration	Endotype discovery and pathway inference using MOFA, iCluster, NMF, CCA or DIABLO	Identifies shared molecular axes and cross-layer biology	Latent factors may not map to clinical endpoints; unstable clusters	Association with stage transition, C-peptide decline or treatment response; replication across cohorts
Network biology integration	Mechanism-focused studies linking genes, proteins, metabolites and pathways	Improves biological interpretability and target discovery	Network priors may be incomplete; difficult clinical translation	Pathway enrichment, network robustness, independent experimental support
Late score-level integration	Stepwise clinical workflow combining GRS, antibodies, OGTT/CGM and targeted omics scores	Interpretable, tolerant of missing data, cost-aware	May miss nonlinear cross-layer interactions	Incremental value over standard markers, net benefit, implementation burden
Longitudinal dynamic modeling	Repeated measures across genetic susceptibility, seroconversion, stage 1 and stage 2	Updates individualized risk over time; reflects disease continuum	Irregular sampling, censoring, missingness and time-varying confounding	Time-dependent AUC/C-index, Brier score, landmark calibration, clinically relevant horizons
Deep learning and AI decision support	Multimodal data with images, CGM streams, omics and clinical features	Captures nonlinear and temporal patterns; supports risk dashboards	Black-box behavior, dataset shift, bias, weak explainability	SHAP/attention explanations, model cards, subgroup performance, prospective silent trial before deployment

#### 4.2.4 Construction of multimodal prediction models

**Figure 1:**
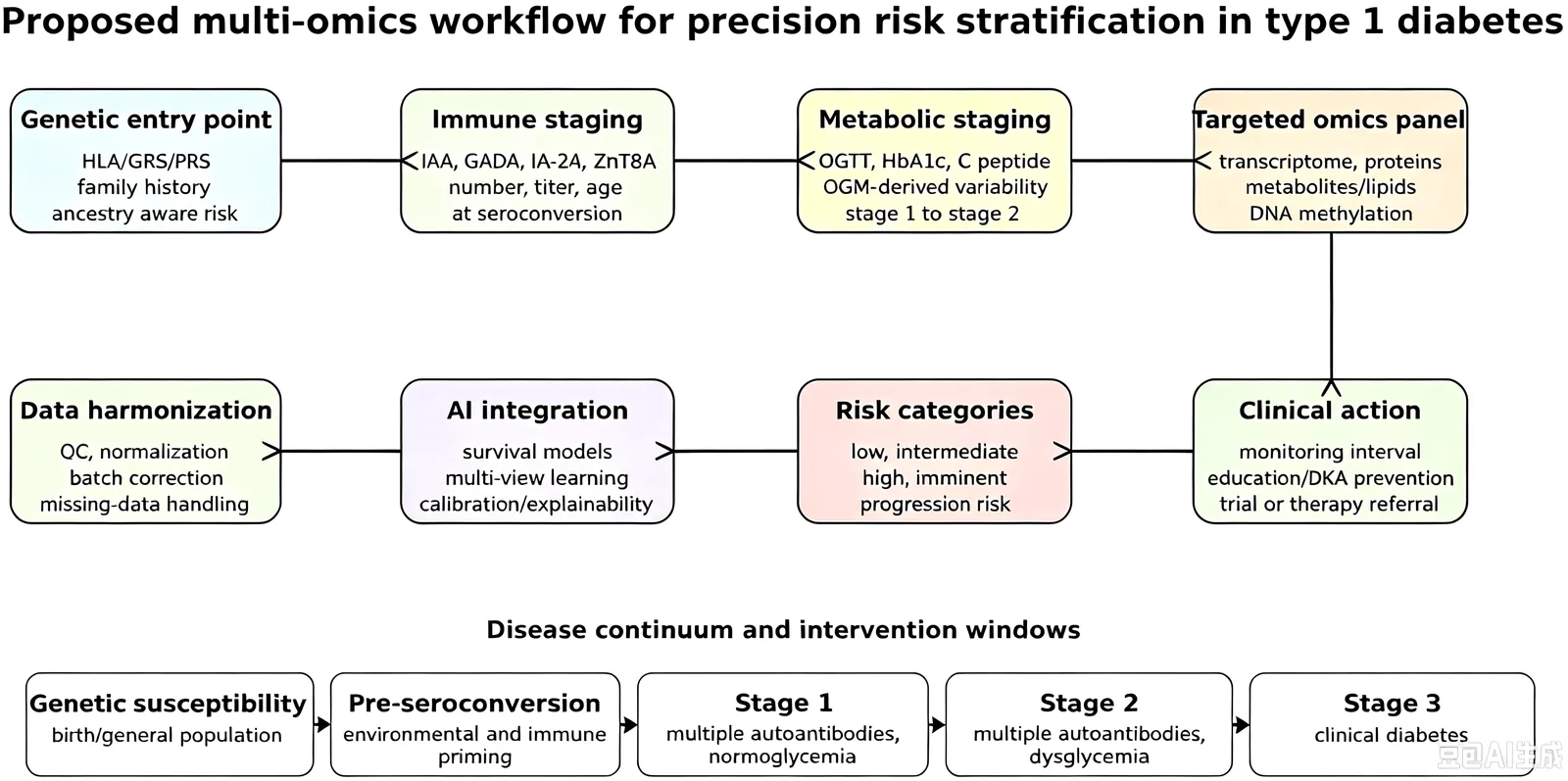
Proposed clinical workflow for integrating conventional T1D staging with targeted multi-omics and AI-based dynamic risk prediction.

One major objective of multi-omics integration is the development of multimodal prediction models that combine inherited genetic risk with dynamic biological and clinical information. By integrating genetic risk scores (GRS), islet autoantibody profiles, transcriptomic and proteomic biomarkers, metabolomic signatures, and conventional clinical indicators such as HbA1c and C-peptide, these models enable continuous risk assessment throughout the disease course rather than relying on single time-point evaluations. Such integrated prediction models are expected to provide more accurate estimates of disease progression and facilitate personalized preventive interventions (57), forming the basis of the proposed stage-adapted clinical workflow illustrated in **Figure 1**.

### 4.3 Representative applications of multi-omics integration

#### 4.3.1 Integration of metabolomics and microbiome profiling

The intestinal microbiota communicates with the host immune system through microbial metabolites, including short-chain fatty acids, bile acids, and amino acid derivatives. Integrating microbiome sequencing with metabolomic profiling enables characterization of the gut–metabolism–immune axis (58). Studies have demonstrated reduced abundance of Bifidobacterium and Parabacteroides together with increased Escherichia-Shigella and Erysipeloclostridium ramosum in individuals with T1D Experimental microbiota transplantation further demonstrated that gut microbial dysbiosis contributes to impaired glucose metabolism and enhanced inflammatory responses, whereas butyrate exerts protective effects on pancreatic β-cell function (59). These findings illustrate how integrated microbiome–metabolome analyses improve mechanistic understanding of disease pathogenesis and identify potential therapeutic targets.

#### 4.3.2 Integration of proteomics and metabolomics

Combining proteomic and metabolomic analyses simultaneously captures functional protein alterations and downstream metabolic phenotypes. A systematic review following PRISMA guidelines summarized 13 serum proteomic studies and identified 266 differentially expressed proteins, including 31 reproducible biomarkers involved in complement activation, lipid metabolism, and immune regulation (32). Integrating these protein biomarkers with metabolomic signatures provides more robust biomarker panels than either modality alone and improves dynamic monitoring of disease progression.

#### 4.3.3 Multi-layer omics integration

A proof-of-concept study by Alcazar et a (60). integrated transcriptomic, proteomic, metabolomic, and lipidomic datasets obtained from first-degree relatives of patients with T1D. This multi-layer analysis identified coordinated activation of CD4^+^ T-cell and macrophage signaling pathways, including NF-κB, TGF-β, VEGF, arachidonic acid metabolism, and arginase pathways. Furthermore, integrated biomarker panels combining microRNAs, proteins, metabolites, and lipids demonstrated greater discriminatory power than individual biomarkers, highlighting the potential of multi-omics integration for comprehensive risk stratification in high-risk populations.

### 4.4 Current challenges and future perspectives

Despite encouraging progress, several important challenges remain before multi-omics integration can be routinely implemented in clinical practice. First, standardized protocols for sample collection, data preprocessing, quality control, and cross-platform normalization remain insufficient, limiting reproducibility across independent studies. Second, the high dimensionality of multi-omics datasets frequently exceeds available sample sizes, increasing the risk of overfitting and reducing model generalizability. Third, prospective validation in large, ethnically diverse cohorts remains limited, and many published biomarkers have not yet undergone external validation. Finally, although AI algorithms substantially improve predictive performance, model interpretability remains an important challenge for clinical implementation.

Future studies should emphasize longitudinal multi-center cohort studies, standardized analytical pipelines, interpretable AI algorithms, and integration with conventional clinical markers. Rather than replacing existing biomarkers, multi-omics integration should complement established clinical risk assessment tools to enable continuous, individualized risk prediction across the entire T1D disease continuum. Ultimately, integrating genomics, transcriptomics, proteomics, metabolomics, epigenomics, microbiome profiling, and clinical phenotyping has the potential to transform T1D management from reactive treatment to proactive precision prevention.

## 5 Conclusion

In summary, T1D is a heterogeneous disease with the joint action of genetic and environmental factors. Traditional single-dimensional evaluation is difficult to meet the needs of accurate early warning and individualized stratification. multi-omic integration can enhance the understanding of the mechanism of diabetes and provide information for personalized treatment methods (3); multi-omic research has identified the complex molecular network behind beta-cell dysfunction, insulin resistance and diabetes complications; by more accurate analysis of the heterogeneity of diabetes, multi-omic integration can achieve targeted intervention and prevention strategies.

Different omics have complementary advantages: genomics lays the genetic foundation, epigenomics reflects environmental-gene interaction, transcriptome and proteomics reflect the dynamics of immunity and islet damage, and metabolomics and microbiology suggest interventional metabolic changes. Combining bioinformatics and AI algorithms, an efficient prediction model can be built to achieve individualized and accurate prevention.

At present, the clinical application of multi-omic learning still faces challenges such as standardization, cost and causal verification. In the future, it is necessary to build a multi-center data platform, develop low-cost testing schemes, and verify the effectiveness of the strategy through forward-looking tests, so as to promote T1D prevention and control from passive treatment to active prevention.
